# A Virulent Systemic Feline Calicivirus Strain Isolated in China Could Produce Broadly Neutralizing Antibodies Against Multiple Strains

**DOI:** 10.1155/tbed/6853477

**Published:** 2025-12-07

**Authors:** Wuchang Heng, Dan Zang, Ruiyu Li, Ruibin Qi, Qian Jiang, Jiasen Liu, Honglin Jia, Hongtao Kang

**Affiliations:** ^1^ State Key Laboratory for Animal Disease Control and Prevention, Harbin Veterinary Research Institute, Chinese Academy of Agricultural Sciences, Harbin, China, hvri.ac.cn; ^2^ Heilongjiang Research Center for Veterinary Biopharmaceutical Technology, Harbin Veterinary Research Institute, Chinese Academy of Agricultural Sciences, Harbin, China, hvri.ac.cn

**Keywords:** feline calicivirus, infectious clone, pathogenicity, vaccine

## Abstract

Feline calicivirus (FCV) is a significant pathogen in cats, with sporadic outbreaks of infections with virulent systemic (VS‐FCV) strains causing significant health problems. Nineteen FCV strains were isolated and identified in China from 2021 to 2022. The nucleotide and amino acid phylogenetic analysis of the VP1 gene showed that 16 strains were GI genotype and three strains were GII genotype. HBDL2 strain was further found to be in the same clade as the reported VS‐FCV SH/2014 strain, with 87.1% nucleotide sequence identity and 93.0% amino acid sequence identity. However, given that phylogenetic and homology analysis alone is insufficient to predict virulence, the pathogenic potential of HBDL2 was subsequently assessed through experimental infection in cats. The results revealed that HBDL2 was able to cause systemic clinical signs and caused severe tracheal and lung damage, with a similar characterization as the VS‐FCV strain. Serum neutralization assays confirmed that HBDL2 elicited broad‐spectrum neutralizing antibodies in cats against multiple FCV strains, including diverse GI and GII genotypes. Notably, neutralizing antibody titers against the VS‐FCV strains were elevated. Additionally, we established an infectious clone of HBDL2 as a critical technical tool. These findings indicate that the HBDL2 strain holds promise for use in vaccine development against FCV infection.

## 1. Introduction

Feline calicivirus (FCV) is part of the Vesivirus genus within the Caliciviridae family and is a common and highly infectious virus affecting cats, posing a threat to their health [1, 2], with its first report dating back to 1957 [3]. FCV can induce a range of clinical manifestations, including upper respiratory signs, oral ulcers, limping syndrome, and virulent systemic disease (VS‐FCV infection) [4–7]. VS‐FCV variants, first described in 2000, are associated with a severe and often fatal multisystemic disease syndrome that differs markedly from classical FCV infection [8]. VS‐FCV infection typically presents with high fever, marked subcutaneous edema notably affecting the head and limbs, alopecia, cutaneous ulceration, and widespread necrotizing lesions involving multiple organs such as the liver, pancreas, and spleen [9–11]. The disease can be fatal in severe cases. This severe clinical presentation poses a substantial threat to feline health and welfare. In recent years, VS‐FCV strains have been reported in multiple regions, indicating an increasing prevalence, a trend that raises significant concern [12–19].

FCV is a nonenveloped, positive‐sense, single‐stranded RNA virus whose genome comprises three open reading frames (ORFs). ORF2 encodes the primary structural protein VP1 [20, 21]. The VP1 gene sequence is commonly used to elucidate phylogenetic relationships among various FCV strains [22, 23]. Calicivirus RNA‐dependent RNA polymerase (RdRp) does not have a sequence correction function and exhibits low fidelity [24, 25], leading to a high mutation rate and diverse genotypes under immune pressure, thus more easily evading the immune clearance of the virus. This extensive diversity presents a major challenge for vaccination. The protection conferred by current commercial vaccines, often based on historical strains like F9, is nonselective and can be unpredictable; while they are effective in reducing disease from classical FCV, they provide variable and often incomplete protection against heterologous, contemporary circulating strains, including both classical and VS‐FCV variants [18, 26]. Therefore, conducting epidemiological investigations and studying the biological characteristics of FCV are of significant importance. Screening currently circulating strains as vaccine candidates may provide a more effective strategy for prevention.

In this study, we isolated and characterized 19 FCV strains from the Hebei, Jiangxi, Shandong, Jilin, and Heilongjiang provinces of China between 2021 and 2022. Pathogenicity analysis of the HBDL2 strain in cats revealed characteristics similar to virulent systemic (VS‐FCV). Furthermore, serum neutralization assays demonstrated that HBDL2 elicited broad‐spectrum neutralizing antibodies against multiple FCV strains of GI and GII genotypes, including several VS‐FCV strains. These findings suggest that the HBDL2 strain holds potential for vaccine development, highlighting its significance for the future prevention and control of FCV outbreaks. Building on this, we established an infectious clone of HBDL2, providing essential technical support for its investigation as a vaccine.

## 2. Materials and Methods

### 2.1. Clinical Samples, Cells, and Viruses

Samples of cat swabs were collected from Hebei, Jiangxi, Shandong, Heilongjiang, and other places and stored at −80°C from 2021. Feline kidney cells (F81) were cultured in Dulbecco’s modified Eagle medium (DMEM, Gibco) with 10% fetal bovine serum (FBS). The reference strains FCV 2280 and F9 were obtained from the American Type Culture Collection (ATCC). All other FCV strains included in this study had been previously isolated, characterized, and preserved in our laboratory [23, 27, 28].

### 2.2. Isolation of the Virus and Plaque Purification

F81 cells were inoculated with the 0.22‐*μ* m membrane‐filtered swab eluate and examined for the appearance of cytopathic effect (CPE) at 12 h intervals. If no CPE was observed after five consecutive blind passages, the sample was considered negative for FCV. For the plaque assay, the FCV isolate was serially diluted 10‐fold and used to infect cell monolayers. Following 1 h of adsorption at 37°C, the inoculum was removed and the cells were overlaid with a 1:1 mixture of 2 x DMEM and 2% low‐melting‐point agarose. After solidification at room temperature, the cells were incubated upside down at 37°C with 5% CO_2_ for 48 h. Individual plaques were then picked using a pipette tip.

### 2.3. Viral Identification

One hundred full‐length FCV genomes were downloaded from the National Center for Biotechnology Information (NCBI) official website and aligned using MegAlign software, and relatively conserved sequence design primers were selected for PCR amplification using KOD‐Plus‐Neo high‐fidelity enzyme (TOYOBO, China). The forward primer: 5′‐GCATGTGCTCAACCTGCGCTAACGT‐3′, and the reverse primer: 5′‐TTTGTGTATGARTAAGGGTC‐3′. The PCR product was sequenced to obtain the VP1 gene sequence.

F81 cells were infected with FCV isolates and monitored for the development of CPE. Expression of FCV VP1 was detected using an indirect immunofluorescence assay (IFA). A mouse monoclonal antibody against the VP1 protein was employed as the primary antibody, followed by incubation with a DyLight 488‐labeled goat anti‐mouse IgG secondary antibody. Green fluorescence signals were visualized using an inverted fluorescence microscope.

### 2.4. Virus Titrations and Growth Curves

We conducted the experiment according to established protocols in our laboratory [29]. The FCV strain was serially diluted 10‐fold, with five replicates per dilution. The diluted virus was inoculated onto F81 cells for 1 h. After removal of the inoculum, the cells were replenished with fresh medium containing 1% FBS and incubated at 37°C for 48 h. The viral titer (TCID_50_) was calculated using the Reed–Muench method based on the observation of CPE.

To establish a viral growth curve, F81 cells were infected with FCV at an MOI of 0.001. Cell lysates and supernatants were collected at 12, 24, 36, 48, 60, and 72 h postinfection for subsequent TCID_50_ assay.

### 2.5. Analysis of FCV VP1 Gene Sequences

In this study, 71 complete genome sequences of FCV were downloaded from the NCBI. The VP1 gene sequences from these genomes, along with the VP1 gene sequence of the isolate obtained in this study, a total of 90 FCV VP1 gene sequences, were selected for analysis. Codon‐based multiple sequence alignments of the nucleotide and amino acid sequences were performed using the MegAlign software. The complete nucleotide and amino acid alignments were then used to conduct phylogenetic analysis with MEGA version 7.0 software. The statistical support for the nodes of the maximum likelihood (ML) phylogenetic tree was evaluated using the bootstrap method with 1000 replicates.

### 2.6. Pathogenicity Experiments of HBDL2 in Cats

Refer to previous procedures for animal experiments [1]. In brief, experimental cats were divided into two groups (*n* = 5 per group): an HBDL2 FCV‐infected group and a DMEM mock‐infected control group. Each cat was inoculated intranasally and ocularly with a total volume of 0.5 mL (0.2 mL per nostril and 0.05 mL per eye) of either the HBDL2 FCV inoculum (viral titer: 2 × 10^8^ TCID_50_/mL) or DMEM. The infection period lasted 15 days. Clinical signs were scored daily (Table S1), and body temperature was measured on days 0, 3, 5, 7, 9, 11, 13, and 15 postinoculation. Oropharyngeal swabs were collected on days 0, 3, 6, 9, 12, and 15 postinoculation to monitor viral shedding. Anticoagulated blood samples were collected on days 0, 3, 6, 9, 12, and 15 postinoculation to quantify viral load by real‐time quantitative PCR. All cats were euthanized on day 15. Tissue specimens from the nasal turbinates, larynx, trachea, and lungs were harvested for viral load detection by real‐time PCR, with lung and tracheal tissues additionally subjected to histopathological analysis. Serum was separated for the neutralization assay. For real‐time quantitative PCR amplification, 20 ng of total RNA from anticoagulated blood and 100 ng from tissue samples were used as templates. The primers and probe used for qPCR detection were as follows: FCV F/R: 5′‐AATTTAATGGTGTGGAGGCGCG‐3′ and 5′‐TGGGGATCCCACCCATAATATTT‐3′, FCV Probe: VIC‐AGCATGTGCTCAACCTGCGCTAACGTGC‐BHQ1.

### 2.7. Virus Neutralization Assays

The HBDL2 FCV‐infected serum was subjected to a two‐fold serial dilution and mixed with an HBDL2 viral strain (2000 TCID_50_/mL) at a 1:1 ratio by vortex oscillation. The mixture was incubated statically at 37°C for 1 h. Subsequently, the mixture was transferred onto F81 cells (90% confluency) seeded in a 96‐well plate. After adsorption for 1 h, the serum–virus mixture was removed and replaced with fresh culture medium. The cells were then incubated at 37°C with 5% CO_2_ for 48 h, after which the presence or absence of CPE was assessed. Each serum dilution was tested in triplicate. A serum dilution was considered to possess complete virus‐neutralizing capacity only if no CPE was observed in all three replicates.

### 2.8. Construction of HBDL2 Infectious Clone and Rescue

We conducted the experiment according to established protocols in our laboratory [29]. The T7 promoter was introduced upstream of the 5′ UTR, and the HDV ribozyme (HDVrz) sequence was inserted downstream of the 3′ UTR of the HBDL2 cDNA genome. The full‐length cassette was then assembled into the pOK12 vector using in vitro homologous recombination. A point mutation was introduced at nucleotide position 136 within the HBDL2 cDNA clone, changing a cytosine (C) to a guanine (G), to serve as a genetic marker for distinguishing the recombinant virus from wild‐type strains. Primers listed in Table S2 were utilized for HBDL2 cDNA cloning. The pOK12 HBDL2 infectious clone was linearized by single‐enzyme digestion, followed by in vitro transcription and capping using T7 RNA polymerase. The resulting RNA was transfected into F81 cells (~90% confluent in 12‐well plates) using Lipofectamine 2000. After 48 h, the transfected cells were subjected to three freeze–thaw cycles at −80°C to harvest the recombinant virus.

### 2.9. Transmission Electron Microscopy

Virus suspensions were applied to copper grids, blotted dry using filter paper, and stained with 2% (w/v) phosphotungstic acid for 45 s. After a second blot‐drying step, grids were air‐dried prior to transmission electron microscopy imaging.

## 3. Results

### 3.1. Virus Identification

**Figure 1:**
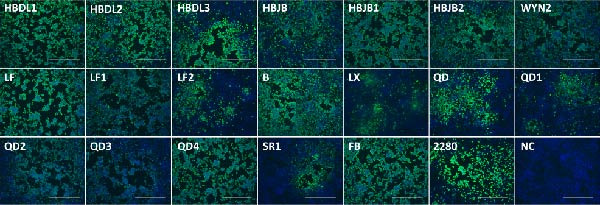
Detection of FCV isolates through IFA. Infection of F81 cells with FCV isolates induced CPE. Expression of the VP1 protein within infected cells was detected by IFA, using cells infected with the 2280 strain as the positive control and uninfected cells as the negative control. Within infected cells, green fluorescence represents the FCV VP1 protein, while blue fluorescence represents the nuclei of both uninfected and cytopathic cells. Scale bar: 400 *μ* m.

In this study, the VP1 antigen was confirmed to be expressed in F81 cells infected with these isolates through the use of monoclonal antibodies specific to the FCV VP1 protein (Figure 1). Collectively, we isolated 19 FCV strains from Hebei, Jiangxi, Shandong, and Heilongjiang provinces in China between 2021 and 2022 (Table S3). The TCID_50_ of the FCV isolates ranged from 10^8.31^ to 10^9.59^.

### 3.2. Sequence Analysis of FCV VP1 Gene

We obtained VP1 gene DNA sequences from 19 FCV isolates by sequencing. The newly determined VP1 gene sequences have been deposited in the GenBank database under accession numbers (PX458761–PX458779). We downloaded the VP1 gene DNA sequences of some GI and GII genotypes FCV and some reported VS‐FCV on the NCBI official website, and built phylogenetic trees using nucleotide and amino acid sequences along with the 19 FCV VP1 gene sequences identified in this study (Figure 2). The phylogenetic tree showed that 16 strains in this study belonged to the GI genotype and three strains belonged to the GII genotype. Currently, GI genotype strains remain dominant and universal worldwide, as also corroborated by our data. It is noteworthy that the GII genotype of FCV is predominantly found in Asia, where it appears to be evolving along a distinct phylogenetic path. This genetic divergence may reduce the efficacy of current FCV vaccines, which were often developed based on strains from other geographical regions. Therefore, surveillance of GII genotype FCV strains is necessary. In addition, we found that HBDL2 and VS‐FCV SH/14 strains belonged to the same clade [14], with the closest genetic distance; the nucleotide sequences showed 87.1% similarity, while the amino acid sequences exhibited 93.0% similarity. In contrast, nucleotide sequence identities with other VS‐FCV strains ranged from 76.6% to 79.2%, and amino acid identities ranged from 85.9% to 89.4%. Compared to vaccine strains, nucleotide and amino acid identities were 77.4%–78.1% and 86.5%–88.3%, respectively. With non‐VS‐FCV strains, nucleotide identities ranged from 75.5% to 78.5%, and amino acid identities ranged from 84.2% to 88.2% (Table S4). Given that phylogenetic relatedness only suggests, but does not definitively prove, a virulent phenotype, we proceeded to evaluate the pathogenicity of HBDL2 through experimental infection in cats.

Figure 2Phylogenetic analysis of the capsid protein VP1 gene of FCV. (A) Nucleotide phylogenetic tree. (B) Amino acid phylogenetic tree. The statistical support for tree nodes was assessed using the bootstrap method with 1000 replicates. Distinct genotypes are clearly labeled and represented by different colors. FCV isolates obtained in this study (brown), vaccine strains (apricot yellow), and virulent strains (red) are color‐coded for clarity. The scale bar indicates the number of nucleotide or amino acid substitutions per site.

**Figure:**
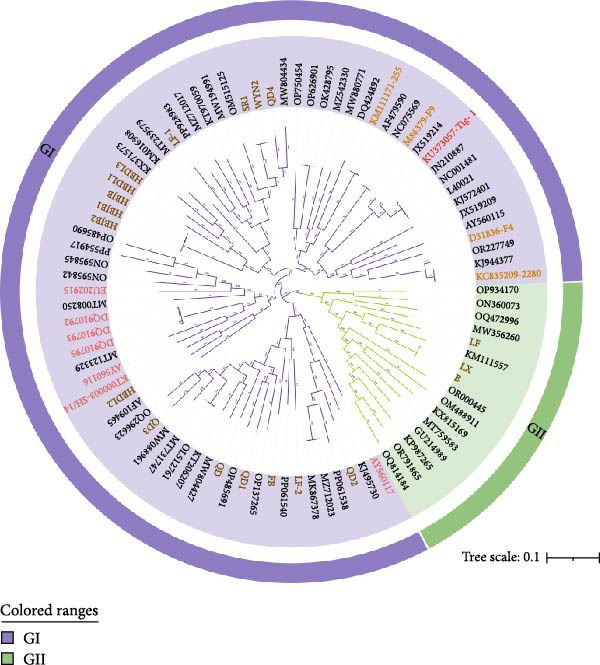
(A)

**Figure:**
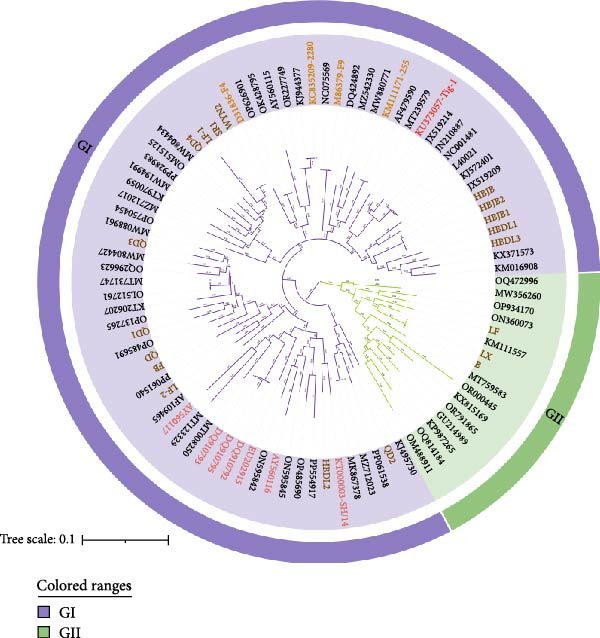
(B)

### 3.3. HBDL2 Infection Leads to Significant Clinical Signs

As indicated by the composite clinical scores (Figure 3A), cats infected with HBDL2 developed severe clinical manifestations compared to controls. Initial signs included pyrexia (Figure 3B), anorexia, and ocular/nasal discharge, with persistent viral shedding detected in throat swabs (Figure 3C). Subsequently, multiple variable‐sized lingual ulcers developed. Ultimately, dyspnea and facial edema emerged, culminating in fatal outcomes in one cat (Figure 3D). On Day 3 postinfection, relatively high viral loads were detected in the blood, which gradually declined to undetectable levels between Days 6 and 15 (Figure 3E), indicating that HBDL2 can induce viremia. Furthermore, HBDL2 infection is capable of invading the nasal turbinates, throat, trachea, and lungs (Figure 3F). Collectively, these findings demonstrate that HBDL2 shares clinicopathological features characteristic of VS‐FCV strains.

Figure 3Pathogenicity of HBDL2. Cats were inoculated with 10^8^ TCID_50_/0.5 mL of strain HBDL2 and monitored for 15 days. (A) Clinical signs were scored daily. (B) Body temperature was measured on days 0, 3, 5, 7, 9, 11, 13, and 15 postinfection. (C) Viral shedding (TCID_50_) in throat swabs was assessed on days 0, 3, 6, 9, 12, and 15 postinfection. (D) Survival rates were recorded. (E) Viral load in blood samples was assessed on days 0, 3, 6, 9, 12, and 15 postinfection. (F) Viral load in nasal turbinates, larynx, trachea, and lungs was assessed on day 15 postinfection. The data are expressed as mean ± standard deviation.

**Figure:**
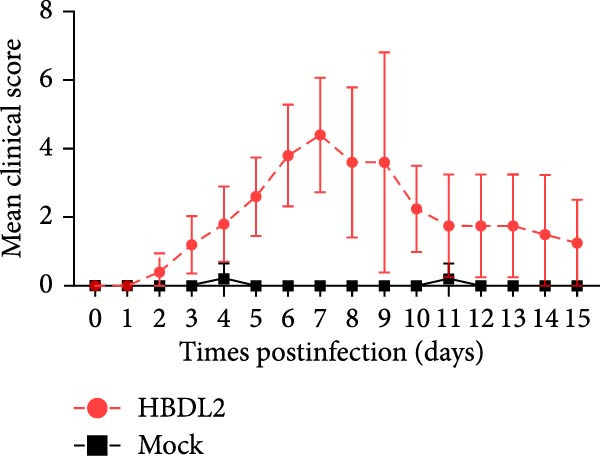
(A)

**Figure:**
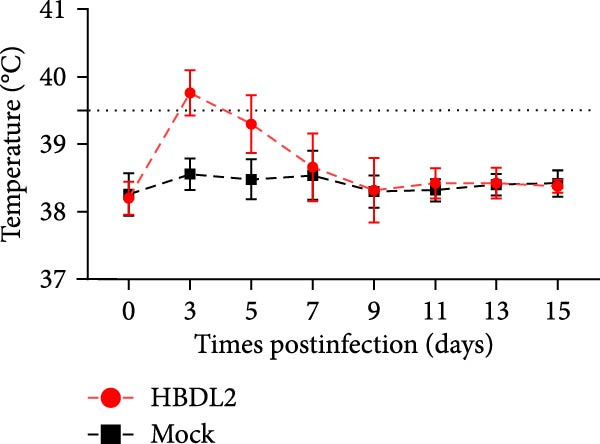
(B)

**Figure:**
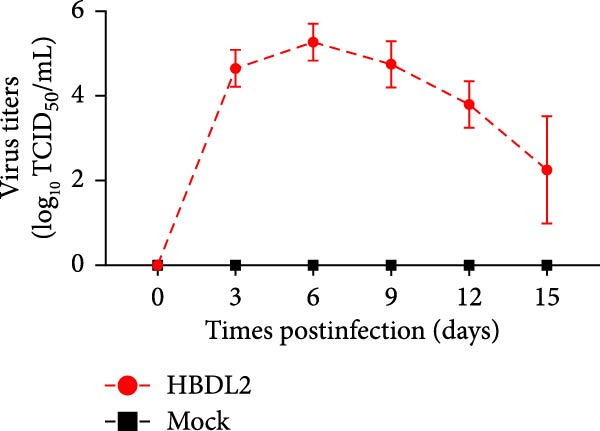
(C)

**Figure:**
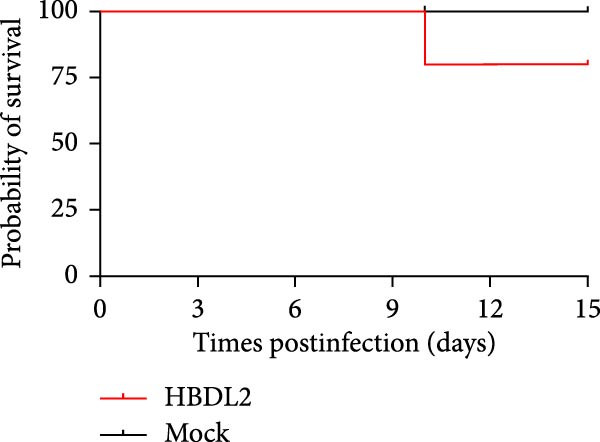
(D)

**Figure:**
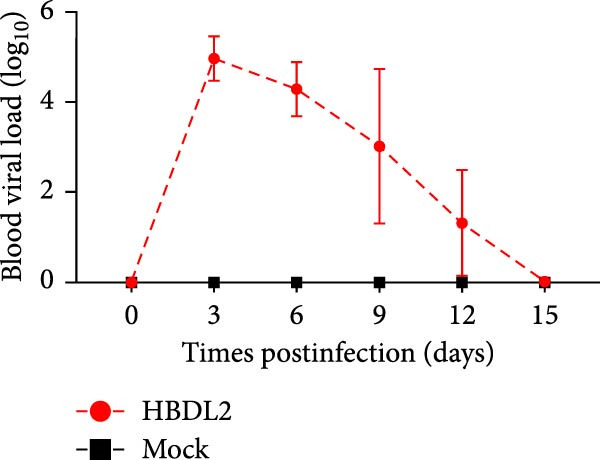
(E)

**Figure:**
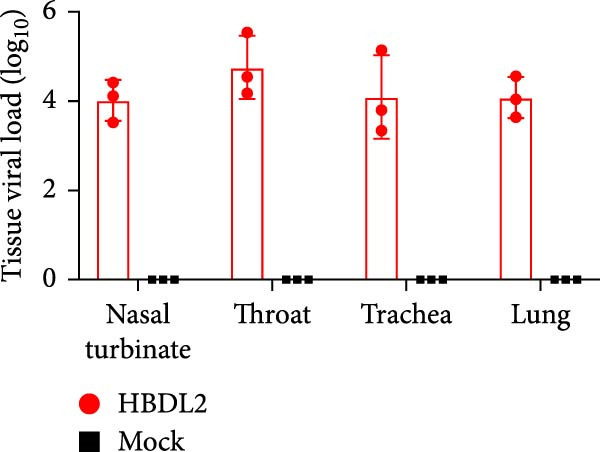
(F)

### 3.4. HBDL2 Infection Can Cause Severe Tracheal and Lung Injury

Following infection with HBDL2 FCV (10^8^TCID_50_/mL), histopathological examination of feline tissues revealed significant submucosal edema in the trachea with focal inflammatory cell infiltration. Pulmonary sections exhibited localized edema, extensive inflammatory cell infiltration, and markedly widened interalveolar septa, demonstrating significantly greater pathological severity compared to control animals (Figure 4A). Histopathological scores are quantified in Figure 4B.

Figure 4Histopathological sections of lungs and trachea from virus‐infected and mock‐infected cats. (A) Lung and tracheal tissues were collected from cats in the virus‐infected group that succumbed to infection on day 10. On day 15, two cats from the virus‐infected group and three cats from the mock‐infected group were euthanized, and their lungs and trachea were harvested for histological examination. Scale bar: 100 or 50 *μ* m. (B) Score histopathological sections from the lung and trachea of cats. 0: normal; 1: mild < 25% involvement; 2: moderate 25%–50%; 3: severe > 50%. The data are expressed as mean ± standard deviation.

**Figure:**
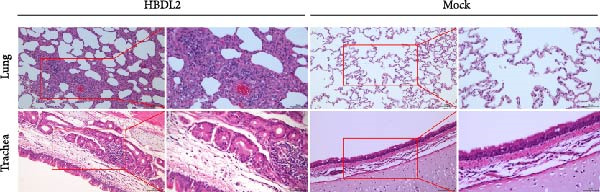
(A)

**Figure:**
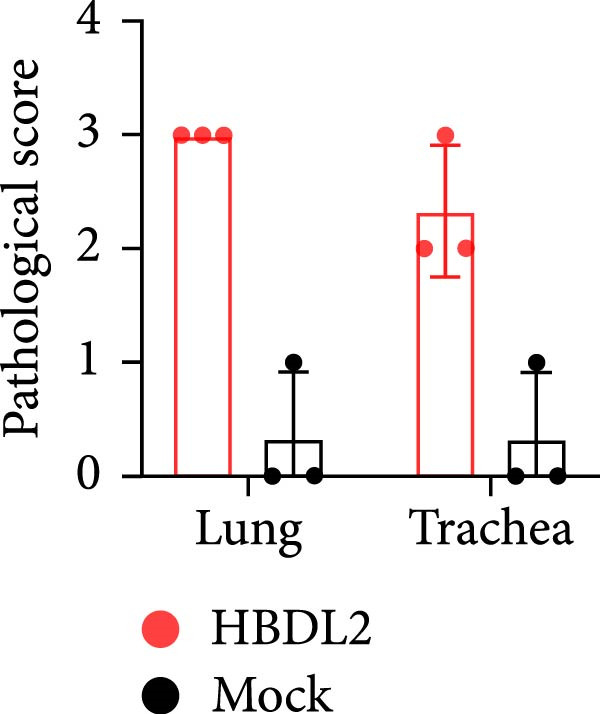
(B)

### 3.5. HBDL2 Produces Broadly Neutralizing Antibodies

Following infection of cats with HBDL2 (10^8^ TCID_50_/mL), venous blood was collected on day 15 to harvest serum, and the titer of serum neutralizing HBDL2 (200 TCID_50_) was determined to be 1:2^10^. This study assessed the ability of HBDL2‐infected sera to neutralize the remaining 18 isolates, along with the classical vaccine strains (F9: Attenuated vaccine strain, 2280: Highly virulent strain for inactivated vaccine) and VS‐FCV strain Tig‐1 (Table 1). The data demonstrated that the neutralizing antibody titers of HBDL2‐infected serum against almost all compared strains reached 1:2^3^ and above, and only the neutralizing titers against QD2 isolates were lower than 1:2^3^. Among them, the neutralizing titers against F9 and 2280 strains were 1:2^7^ and 1:2^6^, respectively. Notably, neutralizing antibody levels against VS‐FCV strains were significantly higher. Specifically, serum neutralization titers against the virulent Tig‐1 strain reached 1:2^8^. The results showed that HBDL2 infection elicited broadly neutralizing antibodies that target several FCV strains.

**Table 1:** Neutralizing antibody titers in serum from HBDL2‐infected cats against heterologous FCV strains.

Viral strain	Genogroup	Origin	Titers
HBDL1^a^	GI	Heilongjiang	2^9^
HBDL2^a^	GI	Heilongjiang	2^10^
HBDL3^a^	GI	Heilongjiang	2^7^
HBJB^a^	GI	Heilongjiang	2^5^
HBJB1^a^	GI	Heilongjiang	2^9^
HBJB2^a^	GI	Heilongjiang	2^7^
WYN2^a^	GI	Heilongjiang	2^7^
LF^a^	GII	Hebei	2^5^
LF‐1^a^	GI	Hebei	2^7^
LF‐2^a^	GI	Hebei	2^6^
B^a^	GII	Hebei	2^3^
LX^a^	GII	Hebei	2^3^
QD^a^	GI	Shandong	2^7^
QD1^a^	GI	Shandong	2^3^
QD2^a^	GI	Shandong	2^2^
QD3^a^	GI	Shandong	2^7^
QD4^a^	GI	Shandong	2^7^
SR1^a^	GI	Jiangxi	2^3^
FB^a^	GI	Jilin	2^5^
Tig‐1^b^	GI	Heilongjiang	2^8^
F9^c^	GI	USA	2^7^
2280^d^	GI	USA	2^6^
SH‐1^b^	GI	Shanghai	2^7^
GZ‐1^b^	GI	Guangdong	2^7^
DL38^e^	GII	Liaoning	2^3^
NJ‐13^f^	GII	Jiangsu	2^3^

^a^Viral isolates from this study.

^b^VS‐FCV strains.

^c^Attenuated vaccine strain.

^d^Highly virulent strain for inactivated vaccine.

^e^Nonvirulent strain.

^f^Respiratory symptoms strains.

### 3.6. Rescue of rHBDL2

The construction of the infectious cDNA clone of rHBDL2 involved inserting a T7 promoter at the 5′ terminus of the FCV genome and adding HDVrz sequence at the 3′ terminus, and introducing a C‐to‐G substitution at nucleotide position 136 (Figure 5A). The viral growth curves of rHBDL2 and WT HBDL2 were evaluated in F81 cells with an MOI of 0.001, and viral titers were measured at 12, 24, 36, 48, 60, and 72 h postinfection. Both recombinant and wild‐type viruses exhibited comparable replication kinetics, with peak titers reaching ~10^9.5^TCID_50_ mL (Figure 5B). Observation by negative‐stain electron microscopy showed that rHBDL2 and WT HBDL2 viral particles both displayed the characteristic morphology of FCV (Figure 5C).

Figure 5Construction of an infectious clone of rHBDL2. (A) Illustrative schematic of the rHBDL2 infectious clone construction. (B) The growth curve for rHBDL2 (MOI = 0.001) in F81 cells was constructed by measuring viral titers (TCID_50_) at 12, 24, 36, 48, 60, and 72 h postinfection. (C) The morphology of rHBDL2 viral particles was examined using transmission electron microscopy. The data are expressed as mean ± standard deviation.

**Figure:**
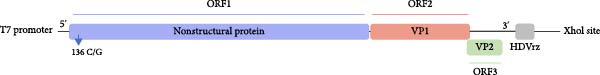
(A)

**Figure:**
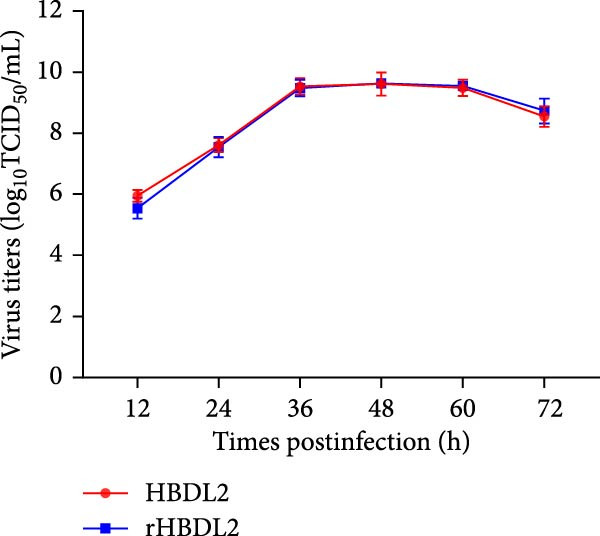
(B)

**Figure:**
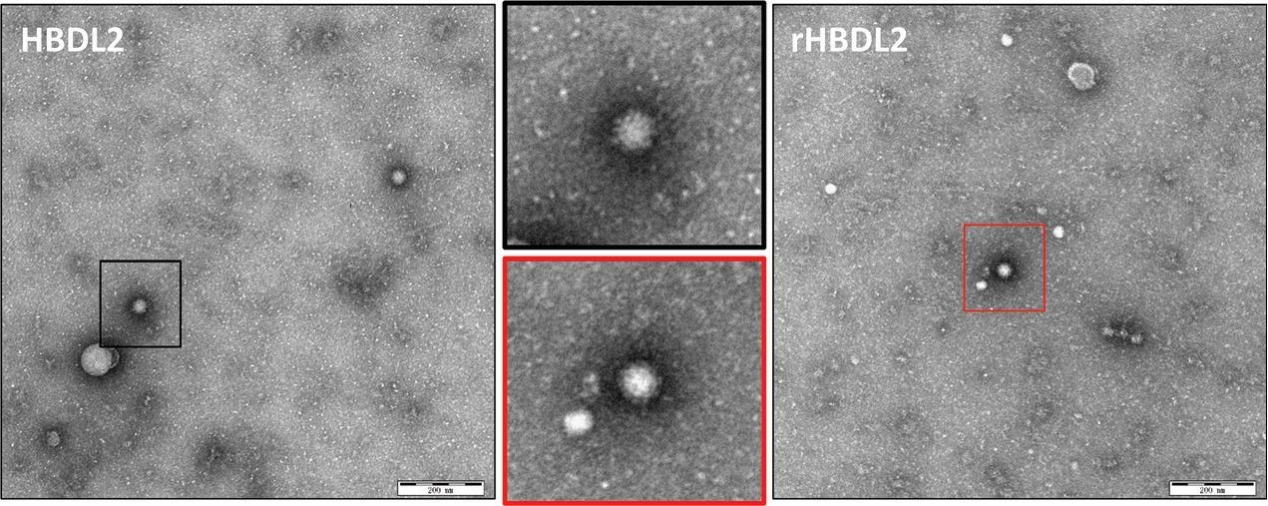
(C)

## 4. Discussion

FCV is among the main infectious agents in cats, causing serious health issues. There are more than 600 million cats globally, and they are one of the main companion animals closely connected to humans [30]. Therefore, in this study, the epidemic surveillance and evolutionary analysis of FCV in different regions of China from 2021 to 2022 aimed to offer data for improved prevention and control measures.

FCV belongs to positive‐sense RNA viruses, and the core of the replication mechanism is the lack of the correction function of virus‐encoded RdRP, which makes genomic mutations frequent [31]. The high mutational variability of FCV and its frequent outbreaks in many regions globally have raised concerns about the effectiveness of existing vaccines. The genetic differences and antigenic variety of FCV make it difficult to effectively control and prevent infection through vaccination, while the continuous emergence of VS‐FCV strains with higher virulence has further challenged the application of existing vaccines. Therefore, improving insights into the epidemiological properties of FCV will help to screen and update effective vaccine candidates against circulating and VS‐FCV strains.

In this study, 19 FCV strains were isolated and identified from China (Hebei, Jiangxi, Shandong, Jilin, and Heilongjiang provinces) from 2021 to 2022. Through phylogenetic analysis of the FCV VP1 gene revealed that among the FCV isolates in this study, 16 belonged to genotype GI and three to genotype GII. Currently, GI is more prevalent worldwide, while GII is primarily distributed in Asia. The emergence of GII FCV warrants increased attention, and conducting regular epidemiological investigations is of significant importance. It is essential to establish animal infection models for GII FCV to evaluate the protective efficacy of current vaccines and candidate vaccine strains against GII variants. Pathogenic studies on GII strains will be the focus of our subsequent research. Furthermore, it was found that the isolate HBDL2 belonged to a clade with the VS‐FCV SH/14 strain; the nucleotide sequences showed 87.1% similarity, while the amino acid sequences exhibited 93.0% similarity. However, no significant difference was observed in the sequence homology between HBDL2 and some other VS‐FCV strains, vaccine strains, as well as non‐VS‐FCV strains. Virulence cannot be reliably predicted based solely on VP1 gene phylogeny and sequence homology. Although the genetic relatedness between HBDL2 and certain VS‐FCV strains (e.g., SH/2014) provides preliminary clues regarding its potential pathogenicity, this association is not definitive. Virulence in FCV is likely a complex polygenic trait, and the precise genetic determinants of VS‐FCV remain elusive. Thus, experimental confirmation of pathogenicity through challenge studies in animal models is indispensable. To definitively assess the virulence of HBDL2, we conducted a pathogenicity assessment in cats. The results revealed that HBDL2 was able to cause systemic clinical signs (including multiple ulcers, respiratory distress, and edema) and viremia in infected cats. Pathological sections showed severe damage to the trachea and lungs of infected cats. Therefore, we believe that isolated HBDL2 has a similar characterization as the VS‐FCV strain. Although the HBDL2 infection model has been established, a limitation remains: the inclusion of a non‐VS‐FCV strain infection group would provide a more direct assessment of the differences in pathogenicity between VS‐FCV and non‐VS‐FCV strains. Notably, we found that HBDL2 elicited broadly neutralizing antibodies against multiple FCV strains, encompassing both GI and GII genotypic FCV isolates. Neutralizing titers against the vaccine strains F9 and 2280 reached 1:2^7^ and 1:2^6^, respectively, while a titer of 1:2^8^ was achieved against the VS‐FCV Tig‐1 strain, despite the Tig‐1 strain’s relatively distant genetic relationship to the HBDL2 strain. This indicates that relying solely on phylogenetic and homology analyses of viral sequences is insufficient for effective vaccine strain selection. Therefore, preliminary screening of candidate vaccine strains should incorporate an assessment of the breadth of neutralizing antibodies induced, followed by further comprehensive evaluation. These results underscore the potential of HBDL2 as a vaccine for combating VS‐FCV and other FCV epidemic strains.

While HBDL2 represents a promising vaccine candidate, we recognize significant challenges in monovalent vaccine development. First, serial in vitro passaging of the virus for vaccine production carries the risk of altering its antigenicity or immunogenicity. Second, widespread use of a single‐strain vaccine may exert substantial selective pressure, potentially facilitating the emergence of vaccine‐escape variants. The HBDL2 infectious clone established in this study provides a key tool to proactively address these challenges. This system enables the reliable rescue of genetically defined viruses with consistent antigenicity, thus allowing essential investigations into genetic stability during serial passages. Moreover, it offers a technical platform for future vaccine development. Previous reports indicate that a vaccine formulation combining two complementary FCV strains enables broad cross‐protection upon heterologous challenge [32]. Ongoing epidemiological surveillance will be critical to identify circulating strains with reduced susceptibility to HBDL2‐induced neutralizing antibodies, with particular attention to FCV of the GII genotype. Such strains could then be incorporated, together with HBDL2, into multivalent vaccine formulations, thereby broadening protection coverage and preemptively mitigating viral escape.

## 5. Conclusion

In summary, we isolated and identified a VS‐FCV strain (HBDL2) from China during 2021–2022. HBDL2 could produce broadly neutralizing antibodies, covering a variety of FCV strains of GI and GII genotypes, and has the potential as a vaccine for the prevention and control of VS‐FCV and common FCV epidemic strains.

## Ethics Statement

All animal experiments in this study were performed according to the Guide for the Care and Use of Laboratory Animals of Harbin Veterinary Research Institute and approved by the Animal Welfare and Ethics Committee of Harbin Veterinary Research Institute (220509‐01‐GR).

## Disclosure

All authors have read and agreed to the published version of the manuscript.

## Conflicts of Interest

The authors declare no conflicts of interest.

## Author Contributions

Conceptualization: Wuchang Heng, Dan Zang, Ruiyu Li, Ruibin Qi, and Qian Jiang performed experiments. Wuchang Heng, Dan Zang, Jiasen Liu, Honglin Jia, and Hongtao Kang analyzed the data. Jiasen Liu, Honglin Jia, and Hongtao Kang conceived, designed, supervised the study, and evaluated all data. Wuchang Heng and Hongtao Kang wrote the paper. Wuchang Heng and Dan Zang contributed equally to this work and are co‐first authors.

## Funding

This study was supported by the National Natural Science Foundation of China (Grant 32470154), the Key Research and Development Program of Heilongjiang Province (Grant 2022ZX02B12), and the Central Public‐interest Scientific Institution Basal Research Fund (Grant 1610302022009).

## Supporting Information

Additional supporting information can be found online in the Supporting Information section.

## Supporting information


**Supporting Information** Table S1: The standard for assessing clinic signs. Table S2: Primers used in HBDL2 cDNA clone. Table S3: FCV isolate information. Table S4: Nucleotide and amino acid sequence identity (%) of FCV HBDL2 and reference FCV strains.

## Data Availability

The data that support the findings of this study are available from the corresponding author upon reasonable request.

## References

[1] Tian J. , Kang H. , and Huang J. , et al.Feline Calicivirus Strain 2280 p30 Antagonizes Type I Interferon-Mediated Antiviral Innate Immunity Through Directly Degrading IFNAR1 mRNA, PLoS Pathogens. (2020) 16, no. 10, 10.1371/journal.ppat.1008944, e1008944. 33075108 PMC7571719

[2] Hofmann-Lehmann R. , Hosie M. J. , and Hartmann K. , et al.Calicivirus Infection in Cats, Viruses. (2022) 14, no. 5, 10.3390/v14050937, 937. 35632680 PMC9145992

[3] Fastier L. B. , A New Feline Virus Isolated in Tissue Culture, American Journal of Veterinary Research. (1957) 18, no. 67, 382–389. 13424932

[4] Pedersen N. , Laliberte L. , and Ekman S. , A Transient Febrile Limping Syndrome of Kittens Caused by Two Different Strains of Feline Calicivirus, Feline Practice. (1983) 13, 26–36.

[5] Dawson S. , Bennett D. , and Carter S. D. , et al.Acute Arthritis of Cats Associated With Feline Calicivirus Infection, Research in Veterinary Science. (1994) 56, no. 2, 133–143, 10.1016/0034-5288(94)90095-7, 2-s2.0-0028390712. 8191001

[6] Binns S. H. , Dawson S. , and Speakman A. J. , et al.A Study of Feline Upper Respiratory Tract Disease With Reference to Prevalence and Risk Factors for Infection With Feline Calicivirus and Feline Herpesvirus, Journal of Feline Medicine and Surgery. (2001) 2, no. 3, 123–133, 10.1053/jfms.2000.0084, 2-s2.0-0034280309. PMC10829116 11716607

[7] Radford A. D. , Addie D. , and Belak S. , et al.Feline Calicivirus Infection. ABCD Guidelines on Prevention and Management, Journal of Feline Medicine and Surgery. (2009) 11, no. 7, 556–564, 10.1016/j.jfms.2009.05.004, 2-s2.0-65749097438. 19481035 PMC11132273

[8] Pedersen N. C. , Elliott J. B. , Glasgow A. , Poland A. , and Keel K. , An Isolated Epizootic of Hemorrhagic-Like Fever in Cats Caused by a Novel and Highly Virulent Strain of Feline Calicivirus, Veterinary Microbiology. (2000) 73, no. 4, 281–300, 10.1016/S0378-1135(00)00183-8, 2-s2.0-0034636393. 10781727 PMC7117377

[9] Hurley K. F. , Pesavento P. A. , Pedersen N. C. , Poland A. M. , Wilson E. , and Foley J. E. , An Outbreak of Virulent Systemic Feline Calicivirus Disease, Journal of the American Veterinary Medical Association. (2004) 224, no. 2, 241–249, 10.2460/javma.2004.224.241, 2-s2.0-0347916960. 14736069

[10] Pesavento P. A. , Maclachlan N. J. , Dillard-Telm L. , Grant C. K. , and Hurley K. F. , Pathologic, Immunohistochemical, and Electron Microscopic Findings in Naturally Occurring Virulent Systemic Feline Calicivirus Infection in Cats, Veterinary Pathology. (2004) 41, no. 3, 257–263, 10.1354/vp.41-3-257, 2-s2.0-2542483566. 15133174

[11] Coyne K. P. , Jones B. R. D. , and Kipar A. , et al.Lethal Outbreak of Disease Associated With Feline Calicivirus Infection in Cats, Veterinary Record. (2006) 158, no. 16, 544–550, 10.1136/vr.158.16.544, 2-s2.0-33646365890. 16632527

[12] Reynolds B. S. , Poulet H. , and Pingret J.-L. , et al.A Nosocomial Outbreak of Feline Calicivirus Associated Virulent Systemic Disease in France, Journal of Feline Medicine and Surgery. (2009) 11, no. 8, 633–644, 10.1016/j.jfms.2008.12.005, 2-s2.0-67650689334. 19201637 PMC11132575

[13] Schulz B. S. , Hartmann K. , and Unterer S. , et al.Two Outbreaks of Virulent Systemic Feline Calicivirus Infection in Cats in Germany, Berliner und Munchener tierarztliche Wochenschrift. (2011) 124, no. 5-6, 186–193. 22059287

[14] Guo H. , Miao Q. , Zhu J. , Yang Z. , and Liu G. , Isolation and Molecular Characterization of a Virulent Systemic Feline Calicivirus Isolated in China, Infection, Genetics and Evolution. (2018) 65, 425–429, 10.1016/j.meegid.2018.08.029, 2-s2.0-85052934695. 30176370

[15] Caringella F. , Elia G. , and Decaro N. , et al.Feline Calicivirus Infection in Cats With Virulent Systemic Disease, Italy, Research in Veterinary Science. (2019) 124, 46–51, 10.1016/j.rvsc.2019.02.008, 2-s2.0-85062369291. 30844542

[16] Park J. , Lee D. , Hong Y.-J. , Hwang C.-Y. , and Hyun J.-E. , Outbreaks of Nosocomial Feline Calicivirus-Associated Virulent Systemic Disease in Korea, Journal of Veterinary Science. (2024) 25, no. 4, 10.4142/jvs.24030. PMC11291428 39083203

[17] Duclos A. A. , Guzmán Ramos P. J. , and Mooney C. T. , Virulent Systemic Feline Calicivirus Infection: A Case Report and First Description in Ireland, Irish Veterinary Journal. (2024) 77, no. 1, 10.1186/s13620-024-00262-3, 7. 38336785 PMC10854173

[18] Schorr-Evans E. M. , Poland A. , Johnson W. E. , and Pedersen N. C. , An Epizootic of Highly Virulent Feline Calicivirus Disease in a Hospital Setting in New England, Journal of Feline Medicine and Surgery. (2003) 5, no. 4, 217–226, 10.1016/S1098-612X(03)00008-1, 2-s2.0-0043170805. 12878149 PMC10822567

[19] Deschamps J.-Y. , Topie E. , and Roux F. , Nosocomial Feline Calicivirus-Associated Virulent Systemic Disease in a Veterinary Emergency and Critical Care Unit in France, Journal of Feline Medicine and Surgery Open Reports. (2015) 1, no. 2, 10.1177/2055116915621581. PMC5362001 28491401

[20] Carter M. J. , Milton I. D. , Turner P. C. , Meanger J. , Bennett M. , and Gaskell R. M. , Identification and Sequence Determination of the Capsid Protein Gene of Feline Calicivirus, Archives of Virology. (1992) 122, no. 3-4, 223–235, 10.1007/BF01317185, 2-s2.0-0026455611. 1731695 PMC7086951

[21] Urban C. , Luttermann C. , and Simon A. E. , Major Capsid Protein Synthesis From the Genomic RNA of Feline Calicivirus, Journal of Virology. (2020) 94, no. 15, 10.1128/JVI.00280-20. PMC7375370 32404528

[22] Fujita S. , Koba R. , and Tohya Y. , Identification of Amino Acid Substitutions Escaping From a Broadly Neutralizing Monoclonal Antibody of Feline Calicivirus, Virus Research. (2022) 318, 10.1016/j.virusres.2022.198848, 198848. 35691421

[23] Yang Y. , Liu Z. , and Chen M. , et al.Classification of Genotypes Based on the VP1 Gene of Feline Calicivirus and Study of Cross-Protection Between Different Genotypes, Frontiers in Microbiology. (2023) 14, 10.3389/fmicb.2023.1226877, 1226877. 37614595 PMC10442547

[24] Lee J.-H. , Chung M. S. , and Kim K. H. , Structure and Function of Caliciviral RNA Polymerases, Viruses. (2017) 9, no. 11, 10.3390/v9110329, 2-s2.0-85033480952, 329. 29113097 PMC5707536

[25] Smertina E. , Urakova N. , Strive T. , and Frese M. , Calicivirus RNA-Dependent RNA Polymerases: Evolution, Structure, Protein Dynamics, and Function, Frontiers in Microbiology. (2019) 10, 10.3389/fmicb.2019.01280, 2-s2.0-85069051592, 1280. 31244803 PMC6563846

[26] Rong S. , Lowery D. , Floyd-Hawkins K. , and King V. , Characterization of an Avirulent FCV Strain With a Broad Serum Cross-Neutralization Profile and Protection Against Challenge of a Highly Virulent Vs Feline Calicivirus, Virus Research. (2014) 188, 60–67, 10.1016/j.virusres.2014.03.007, 2-s2.0-84903738370. 24685673

[27] Tian J. , Liu D. , and Liu Y. , et al.Molecular Characterization of a Feline Calicivirus Isolated From Tiger and its Pathogenesis in Cats, Veterinary Microbiology. (2016) 192, 110–117, 10.1016/j.vetmic.2016.07.005, 2-s2.0-84978252766. 27527772

[28] Yang Y. , Chen M. , and Liu Z. , et al.Analysis of Genetic Diversity Based on Sequences of Feline Calicivirus Strains Isolated in China, Transboundary and Emerging Diseases. (2025) 2025, 10.1155/tbed/9924540, 9924540. 40873503 PMC12380517

[29] Heng W. , Zhang D. , and Li R. , et al.A Novel Replication-Deficient FCV Vaccine Provides Strong Immune Protection in Cats, Journal of Virology. (2025) 99, no. 8, 10.1128/jvi.00093-25, e0009325. 40626663 PMC12363202

[30] Hosie M. J. and Hofmann-Lehmann R. , Special Issue: Viral Infections in Companion Animals, Viruses. (2022) 14, no. 2, 10.3390/v14020320, 320. 35215913 PMC8878503

[31] Castro C. , Arnold J. J. , and Cameron C. E. , Incorporation Fidelity of the Viral RNA-Dependent RNA Polymerase: A Kinetic, Thermodynamic and Structural Perspective, Virus Research. (2005) 107, no. 2, 141–149, 10.1016/j.virusres.2004.11.004, 2-s2.0-11844283982. 15649560 PMC7125856

[32] Poulet H. , Brunet S. , Leroy V. , and Chappuis G. , Immunisation With a Combination of Two Complementary Feline Calicivirus Strains Induces a Broad Cross-Protection Against Heterologous Challenges, Veterinary Microbiology. (2005) 106, no. 1-2, 17–31, 10.1016/j.vetmic.2004.12.010, 2-s2.0-14544272787. 15737470

