# Supplementary material for: A Virulent Systemic Feline Calicivirus Strain Isolated in China Could Produce Broadly Neutralizing Antibodies Against Multiple Strains
Source: Transbound Emerg Dis. 2025 Dec 7;2025:6853477. doi: 10.1155/tbed/6853477 (PMC12697810; doi:10.1155/tbed/6853477)
Supplement: Supplementary file 1 — Supporting Information Table S1: The standard for assessing clinic signs. Table S2: Primers used in HBDL2 cDNA clone. Table S3: FCV isolate information. Table S4: Nucleotide and amino acid sequence identity (%) of FCV HBDL2 and reference FCV strains. [file TBED-2025-6853477-s001.docx]

**Table S1. The standard for assessing clinic signs.**

| **Score** | **Depression and anorexia** | **Oral cavity signs** | **Respiratory signs** | **Ocular discharges** | **Lameness** |
| --- | --- | --- | --- | --- | --- |
| **0** | no signs | no signs | no signs | no signs | no signs |
| **1** | depression | one little ulcer spot (diameter*<*0.5 cm) | sneezing (1–2 times  per 10 min) | clear secretion  (one eye) | walking posture deformation and able to bear weight on the affected foot |
| **2** | 1/3-1/2 food intake | 2–3 little ulcer spots (diameter*<*0.5 cm) | sneezing (1–3 time  per 5 min) | clear secretion (two eyes) | reluctance to bear weight on the affected foot and unwillingness to place weight on the affected limb, sitting with the limb off the ground |
| **3** | apastia | big ulcer spots (diameter*>*1 cm) | mouth breathing and wheezing | purulent secretion | disable to bear weight on the affected foot, and trouble walking and rising |

**Table S2. Primers used in HBDL2 cDNA clone**

| **Primer** | **Sequence (5'–3')** | **Fragment size (bp)** |
| --- | --- | --- |
| HBDL2-1-F | ACCTAATACGACTCACTATAGTAAAAGAAATTTGAGACAA | 2459 |
| HBDL2-1-R | TGTTGATTGGCGGGTAGTTCATCC |  |
| HBDL2-2-F | GAACTACCCGCCAATCAACATGTGG | 2892 |
| HBDL2-2-R | AACTTCGAACACATCACAGTGGAGG |  |
| HBDL2-3-F | ACTGTGATGTGTTCGAAGTTTGAGC | 2506 |
| HBDL2-3-R | GCCCAGGTCGGACCGCGAGGAGGTGGAGATGCCATGCCGACCCTTTTTTTTTTTTTTTTTTTTTTTTTTTTTTTTTTTTTTTTTTTTTTCCCTGGGGTTAGGCGCAGGTGCGGC |  |
| pOK12-1-F | CCTCGCGGTCCGACCTGGGCATCCGAAGGAGGACGCACGTCCACTCGGATGGCTAAGGGAGGGCGctcgagGcggccgctctagaactagtggatcgatcccca | 2141 |
| pOK12-1-R | CTATAGTGAGTCGTATTAGGTACCTtcgaggcctcggactagtggcgtaatc |  |
| 136 C/G-F | AGTACTCTTTAAACAAGCTGGAGCGCGCTATGCGTCTCGA | 9957 |
| 136 C/G-R | TCGAGACGCATAGCGCGCTCCAGCTTGTTTAAAGAGTACT |  |

**Table S3. FCV isolate information**

| **Number** | **Genogroup** | **Isolate** | **Origin** | **Year** |
| --- | --- | --- | --- | --- |
| 1 | GI | HBDL1 | Harbin, Heilongjiang | 2021 |
| 2 | GI | HBDL2 | Harbin, Heilongjiang | 2021 |
| 3 | GI | HBDL3 | Harbin, Heilongjiang | 2021 |
| 4 | GI | HBJB | Harbin, Heilongjiang | 2021 |
| 5 | GI | HBJB1 | Harbin, Heilongjiang | 2021 |
| 6 | GI | HBJB2 | Harbin, Heilongjiang | 2021 |
| 7 | GI | WYN2 | Harbin, Heilongjiang | 2021 |
| 8 | GII | LF | Langfang, Hebei | 2021 |
| 9 | GI | LF-1 | Langfang, Hebei | 2021 |
| 10 | GI | LF-2 | Langfang, Hebei | 2021 |
| 11 | GII | B | Langfang, Hebei | 2022 |
| 12 | GII | LX | Langfang, Hebei | 2022 |
| 13 | GI | QD | Qingdao, Shandong | 2021 |
| 14 | GI | QD1 | Qingdao, Shandong | 2021 |
| 15 | GI | QD2 | Qingdao, Shandong | 2021 |
| 16 | GI | QD3 | Qingdao, Shandong | 2021 |
| 17 | GI | QD4 | Qingdao, Shandong | 2021 |
| 18 | GI | SR1 | Shangrao, Jiangxi | 2021 |
| 19 | GI | FB | Yanbian, Jilin | 2021 |

**Table S4. Nucleotide and amino acid sequence identity (%) of FCV HBDL2 and reference FCV strains**

| **Strains** | **Genbank accession numbers** | **ORF2（VP1）** | |
| --- | --- | --- | --- |
|  |  | **nt** | **aa** |
| UTCVM-H1^1^ | AY560116 | 79.0 | 89.4 |
| UTCVM-H2^1^ | AY560117 | 77.5 | 86.1 |
| FCV-George^1^ | DQ910792 | 79.2 | 89.2 |
| FCV-Jengo^1^ | DQ910793 | 79.2 | 88.9 |
| FCV-Kaos^1^ | DQ910795 | 78.9 | 88.3 |
| 4b^1^ | EU202915 | 78.3 | 88.9 |
| Tig-1^1^ | KU373057 | 76.6 | 88.2 |
| SH/14^1^ | KT000003 | 87.1 | 93.0 |
| 2280^1, 2^ | KC835209 | 78.3 | 85.9 |
| F9^2^ | M86379 | 77.4 | 86.5 |
| 255^2^ | KM111171 | 78.0 | 88.3 |
| F4^2^ | D31836 | 77.9 | 87.6 |
| 2024^2^ | AF479590 | 78.1 | 88.3 |
| DL31^3^ | MW804427 | 78.5 | 87.1 |
| DL39^3^ | MW804430 | 78.1 | 87.7 |
| DL38^3^ | MW804429 | 75.1 | 84.5 |
| QD-7^3^ | ON360073 | 75.5 | 84.2 |
| QD-164^3^ | ON360072 | 77.7 | 88.2 |

1: VS-FCV strains, 2: Vaccine strains, 3: Non-VS-FCV strains
